# The spectrum of imaging manifestations of Gorham–Stout disease: a novel dynamic contrast-enhanced MR lymphangiography

**DOI:** 10.1186/s13023-023-02704-7

**Published:** 2023-04-26

**Authors:** Yuna Lee, Seunghyun Lee, Saebeom Hur, Yun Soo Jeong, Dong In Suh, Jangsup Moon, Man Jin Kim, Young Hun Choi, Jung-Eun Cheon

**Affiliations:** 1 Department of Radiology, Seoul National University Hospital, 101 Daehak-ro, Jongno-gu, Seoul, 03080 Republic of Korea; 2 Department of Radiology, Seoul National University College of Medicine, 103 Daehak-ro, Jongno-gu, Seoul, 03080 Republic of Korea; 3 Department of Pediatrics, Seoul National University Hospital, 101 Daehak-ro, Jongno-gu, Seoul, 03080 Republic of Korea; 4 Department of Neurology, Laboratory for Neurotherapeutics, Biomedical Research Institute, Seoul National University Hospital, 101 Daehak-ro, Jongno-gu, Seoul, 03080 Republic of Korea; 5 Department of Laboratory Medicine, Seoul National University Hospital, 101 Daehak-ro, Jongno-gu, Seoul, 03080 Republic of Korea; 6 Institute of Radiation Medicine, Seoul National University Medical Research Center, 101 Daehak-ro, Jongno-gu, Seoul, 03080 Republic of Korea

**Keywords:** Gorham–Stout disease, Osteolysis, Lymphatic malformation, Angiomatous lesion, Dynamic contrast-enhanced magnetic resonance lymphangiography

## Abstract

**Background:**

To describe the radiological features of Gorham–Stout disease (GSD) as evaluated using plain radiography and dynamic contrast-enhanced magnetic resonance lymphangiography (DCMRL) imaging techniques.

**Methods:**

Clinical and conventional imaging data were retrospectively reviewed for 15 patients with GSD between January 2001 and December 2020. After December 2018, DCMRL examinations were performed for lymphatic vessel evaluation in patients with GSD and reviewed in four patients.

**Results:**

The median age at diagnosis was 9 years (range: 2 months–53 years). The clinical manifestations were dyspnea in seven patients (46.7%), sepsis in 12 (80.0%), orthopedic problems in seven (46.7%), and bloody chylothorax in seven (46.7%). The common sites of osseous involvement were the spine (73.3%) and pelvic bone (60.0%). Among the non-osseous involvements, peri-osseous infiltrative soft-tissue abnormalities adjacent to the area of bone involvement were the most common (86.7%), followed by splenic cysts (26.7%) and interstitial thickening (26.7%). DCMRL demonstrated weak central conducting lymphatic flow in two patients with abnormal giant tortuous thoracic ducts and no flow in one patient. All patients who underwent DCMRL in this study presented with altered anatomical lymphatics and functional flow with collateralization.

**Conclusion:**

DCMRL imaging and plain radiography are very useful for determining the extent of GSD. DCMRL is a novel imaging tool for the visualization of abnormal lymphatics in patients with GSD, which helps in further treatment. Therefore, in patients with GSD, it might be necessary to obtain not only plain radiographs but also MR and DCMRL images.

**Supplementary Information:**

The online version contains supplementary material available at 10.1186/s13023-023-02704-7.

## Background

Gorham–Stout disease (GSD) is a rare disease characterized by lymphatic vascular channel proliferation that induces soft-tissue changes and progressive osteolysis [1–4]. Decades after Gorham and Stout first reported the clinical features and pathological descriptions of the disease with osteolysis and vessel endothelial changes in 1955, GSD remains challenging to diagnose [2, 5].

One histopathological basis of GSD is that abnormally proliferated lymphatic vessels result in flow reflux into the bone marrow cavity, resulting in weakened bone strength and bone destruction [6]. Multiple extraosseous symptoms may occur, such as the formation of a low-flow lymphatic vessel in one soft tissue or abnormal fluid retention due to fluid exudation into the body cavity caused by the proliferation of lymphatic vessels [6, 7]. Therefore, it is necessary to identify features that can show not only the osteolytic changes in plain radiographs that are common in patients with GSD, but also lymphatic vessel proliferation.

Previous reports have attempted to elucidate the imaging diagnosis for GSD based on bone and soft-tissue changes found on magnetic resonance imaging (MRI) along with multiple osteolysis seen on plain radiographs [4, 5, 7–9]. However, there were limitations due to the rare incidence of GSD and lack of methods to visualize the lymphatic structure. Recently, intranodal dynamic contrast-enhanced MR lymphangiography (DCMRL) by injecting a gadolinium contrast agent into bilateral inguinal lymph nodes and imaging lymphatics was introduced as a visualization tool for lymphatic abnormalities [10].

In patients with GSD, it might be necessary to obtain not only plain radiographs but also DCMRL images. This study aimed to describe the significant radiological features of GSD.

## Methods

This study was approved by our institutional review board. The requirement for informed consent was waived due to the retrospective nature of the study.

From January 2001 to December 2020, patients with GSD were selected based on the search results for the diagnosis name “Gorham–Stout disease” in the Seoul National University Hospital Patient Research Environment system. Patients were selected from this cohort if one of the following imaging techniques was available: plain radiography, computed tomography (CT), MRI, DCMRL, or digital subtraction lymphangiography.

Clinical data were collected from the initial diagnosis of each patient during the follow-up period. Data such as the age at diagnosis, initial symptoms, history of sepsis, and history of surgery were collected, and the fluid nature was recorded as chylous, bloody, or mixed effusion. The tissue specimen was reviewed for confirmation of GSD based on the presence of dilated thin-walled blood vessels and the results of immunohistochemical staining.

Plain radiographic findings were recorded as the number and distribution of osteolysis involvement, which included the vertebrae, cranium, rib, pelvis, and upper/lower extremity bones. The presence of progressive osteolysis and scoliosis were also recorded during the follow-up period. Conventional CT or MRI examinations were performed for regional evaluation of the affected bony structures or non-osseous components. After December 2018, intranodal DCMRL examinations were performed to evaluate lymphatic vessels in patients with GSD. Immediately prior to DCMRL, ultrasound-guided puncture of bilateral lymph nodes were performed using a 26-gauge needle, followed by the cannulation [11, 12]. The detailed MR sequences are listed in Table 1. The intranodal DCMRL examination sequences included T2- and T1-weighted axial images from the thoracic duct outlet to the pelvic cavity. Dynamic contrast-enhanced T1-weighted coronal images were acquired to view the distribution of the contrast agent administered through inguinal lymph nodes. Maximum intensity projections (MIPs) of contrast-enhanced T1-weighted coronal images were reconstructed to show only the lymphatic vessels containing the contrast agent. Intranodal DCMRL findings included the presence of central conducting lymphatics and abnormal lymphatics in the involved bones. Patients who underwent digital subtraction lymphangiography were evaluated to confirm DCMRL findings. All statistical analyses were performed using Microsoft Excel for Mac (version 16.3).

**Table 1:** Clinical characteristics of Gorham–Stout disease

No.	Gender/Age (years)	Onset (years)	Chief Complaint	Sepsis History	Biopsy	Effusion	Edema	Lymphatic Imaging
1	M/3	2 months	Buttock asymmetry	–	Ileum, left	Chylothorax (Bloody)	Trunk edema	DCMRL
2	M/14	3	Dyspnea, Leg swelling	Cellulitis	Ileum, right	Chylothorax (Bloody)	Trunk/Leg edema	DCMRL
3	M/15	4	Dyspnea	–	Ileum, right	Chylothorax (Bloody)	–	DCMRL
4	M/18	8	Dyspnea	Pneumonia	N/A	Chylothorax (Bloody)	Trunk edema	
5	M/19	10	Finger pain	Cellulitis	5th finger, left	–	–	
6	M/19	1	Dyspnea	Pneumonia	7th rib, right	Chylothorax (Bloody)	Trunk edema	
7	M/22	8	Dyspnea	Pneumonia	N/A	Chylothorax (Bloody)	Trunk edema	
8	M/27	7	Headache, fever	Meningitis, CSF rhinorrhea	Sphenoid sinus	–	–	
9	M/27	9	Arm weakness	Osteomyelitis	Humerus, right	–	–	
10	M/31	14	Dyspnea	Pneumonia, Osteomyelitis	Femur, left	Chylothorax (Bloody)	Trunk edema	DCMRL
11	F/31	19	Ileum fracture	Cellulitis	Ileum, left	–	Trunk edema	
12	F/47	30	Pelvic pain	Osteomyelitis	Ileum, unspecified	–	–	
13	M/48	42	Pelvic pain	Osteomyelitis	Ileum, left	Pleural effusion (unspecified)	Trunk/Leg edema	
14	F/50	13	Femur fracture	Peritonitis, Cellulitis	Femur, left	Ascites	Leg edema	
15	F/57	53	Left hip pain	–	Ileum, unspecified	–	–	

CSF: cerebrospinal fluid; DCMRL: dynamic contrast-enhanced magnetic resonance lymphangiography; M: male; F: female; N/A: not applicable

## Results

### Clinical characteristics

A total of 15 patients [4 (26.7%) females and 11 (73.3%) males] were included in this study, 13 (86.7%) of whom had histopathologically confirmed bone lesions (e.g., ileum, rib, pelvic bone, vertebra, and finger), but the records of two patients were unavailable (Table 2). The median age at diagnosis was 9 years (range: 2 months–53 years). Further, 12 (80.0%) patients had pediatric-onset disease (< 20 years old).

**Table 2:** Dynamic contrast-enhanced magnetic resonance lymphangiography protocol

Sequence	Plain	TR/TE (msec)	Flip angle (degrees)	Matrix	Slide Thickness (mm)	ETL	Bandwidth (Hz)	Time (s)
T2WI BLADE TSE	Axial	5770/101	129	320 × 320	8	25	260	180
Pre-contrast 3D GRE T1WI (Radial VIBE)	Axial	3.9/1.8	11	256 × 256	3	1	501	180
Pre-contrast 3D GRE T1WI (VIBE)	Coronal	3.3/1.2	30	320 × 114	1	1	504	40
GBCM 7.5 mL in the bilateral inguinal lymph nodes, slowly injected by hand (approximately 0.5-1 mL/min)								
Post-contrast 3D GRE T1WI (VIBE) every minute	Coronal	3.3/1.2	30	320 × 114	1	1	504	40
Post-contrast 3D GRE T1WI (Radial VIBE)	Axial	3.9/1.8	11	256 × 256	3	1	501	180

GBCM: gadolinium-based contrast medium; ETL: echo train length; GRE: gradient echo; TE: echo time; TR: repetition time; TSE: turbo spin echo

The clinical presentations of GSD depend on the site and extent of involvement. The most common symptom was dyspnea in seven patients (46.7%), followed by pain in four and pathological fracture in two. 12 (80.0%) patients had a history of sepsis caused by pneumonia, cellulitis, peritonitis, or osteomyelitis. Seven patients (46.7%) underwent orthopedic surgical treatment for bone lesions and pathological fractures. Seven patients (46.7%) had chylous and bloody pleural effusions, one had ascites, and none had significant pericardial effusions. Nine patients (60.0%) had soft-tissue edema involving the trunk and lower extremities.

### Image characteristics

**Fig. 1:**
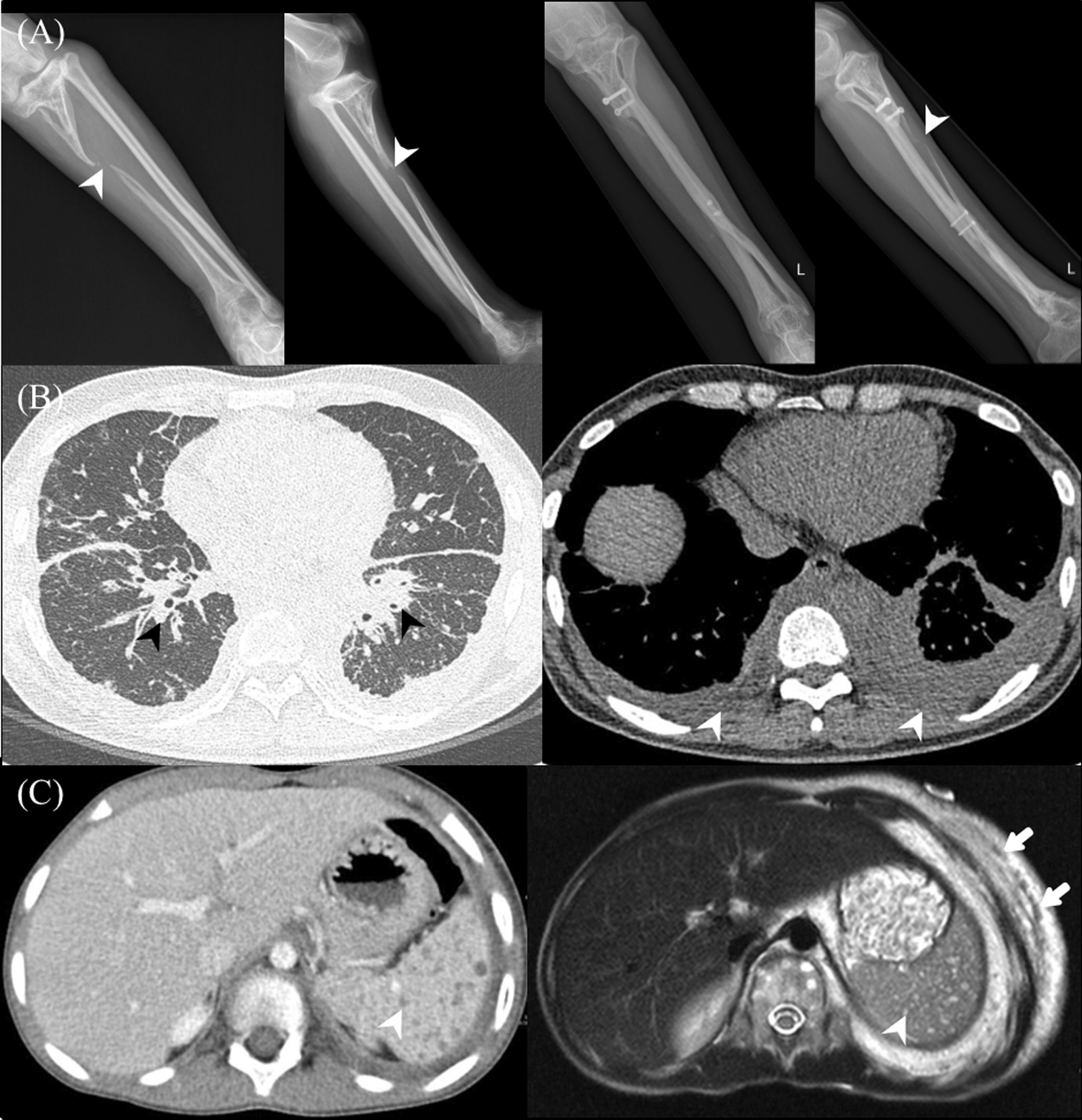
Conventional image characteristics in Gorham–Stout disease. **A** Plain radiograph images of the left lower leg of a 31-year-old man with Gorham–Stout disease (GSD). A vanishing appearance of left tibial proximal metaphysis and diaphysis (arrowhead) with compensatory hypertrophy of the left fibula is seen (first and second columns). A 7-year follow-up plain radiograph of the left lower leg after surgical correction of deformity showing further progression of the osteolysis (arrowhead) despite the operation (third and fourth columns). **B** Chest computed tomography (CT) images of a 48-year-old man diagnosed with GSD. On axial non-contrast chest CT images, interlobular septal line thickening and bronchovascular bundle thickening (black arrowheads) were observed in both lungs, with diffuse pleural thickening (white arrowheads) of the bilateral hemithorax. **C** CT and magnetic resonance images of a 22-year-old man diagnosed with GSD. On axial contrast-enhanced abdominal CT images (first column), multiple small, low-attenuated nodular lesions (arrowhead) were observed in the spleen, but there was no splenomegaly. On axial T2-weighted images (second column), these lesions showed high T2 signal intensities (arrowhead). These could be considered as the splenic manifestation of GSD. Further, there were prominent soft-tissue lesions in the anterior mediastinum and collaterals along the left chest wall (arrows)

Osseous lesions were observed on plain radiography in all patients. The distribution of bone involvement on plain radiographs is shown in Additional file 1: Table S1. All patients had osseous lesions characterized by lytic, lattice-like lucent lesions on plain radiographs. The vertebrae (73.3%) were the most common site of osseous involvement, especially the lumbar vertebrae (66.7%), followed by the pelvic bone (60.0%), lower extremity (40.0%), and rib (40.0%). 14 patients (93.3%) showed progressive osteolysis during the follow-up period, and nine patients (60.0%) showed scoliotic curvature. Osseous lesions were occasionally associated with pathological fractures (Fig. 1A).

**Fig. 2:**
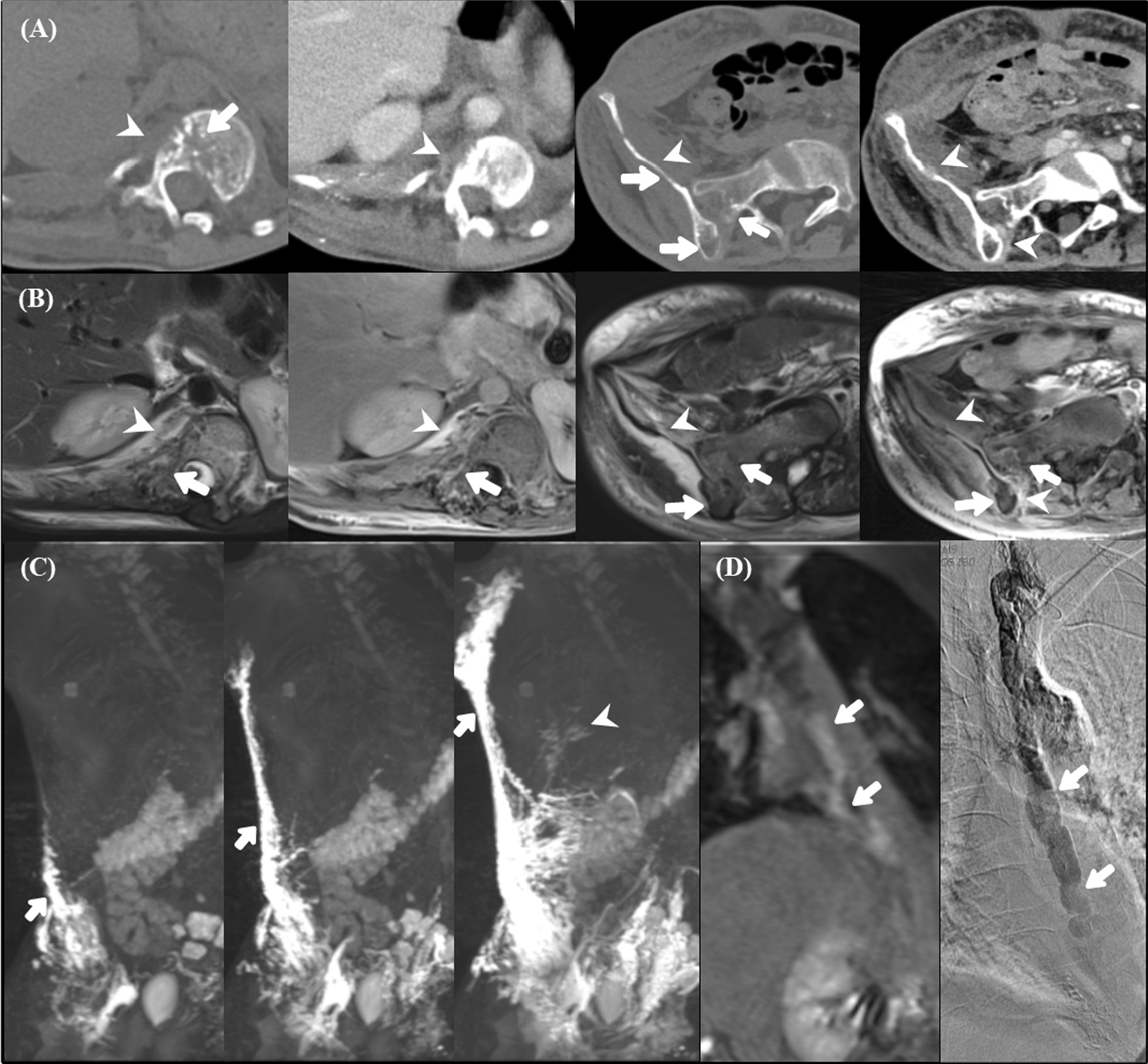
A 14-year-old male with Gorham–Stout disease. A 14-year-old man diagnosed with Gorham‒Stout disease presented with dyspnea related to pleural effusion on the right side. **A** Non-contrast (first and third columns) and contrast-enhanced (second and fourth columns) computed tomography (CT) images showing osteolytic lesions (arrows) and thickened, enhancing infiltrative soft-tissue lesions (arrowheads) at the lower thoracic vertebra and ileum. Extensive soft-tissue edematous changes were also noted in the right abdominal wall. **B, C** Dynamic contrast-enhanced magnetic resonance lymphangiography (DCMRL) consisting of axial T2-weighted, axial and coronal contrast-enhanced T1-weighted, and maximum intensity projection (MIP) reconstruction images. **B** A T2 high signal and contrast-enhanced infiltrative soft-tissue lesion (arrowheads) along the pleura and a contrast-enhancing intraosseous lesion (arrows) are seen (first and second columns). Similar signal-changed soft-tissue lesions are observed along the iliac bone (arrowheads) and the intraosseous lesions (arrows) on the DCMRL image of the right iliac bone (third and fourth columns). These lesions could not delineate the vascular components in those areas on CT images. **C** MIP images (first, second, and third columns) show extensive collateral lymphatic vascular channels along the right abdominal and chest walls (arrows) and faint intercostal lymphatics in the right lower hemithorax (arrowhead). **D** A faintly filled, large abnormal thoracic duct is visible on the T1-weighted coronal image before MIP reconstruction, suggesting weak central conducting lymphatic flow (arrows). The giant thoracic duct (arrows) is confirmed using digital subtraction angiography (right column)

**Fig. 3:**
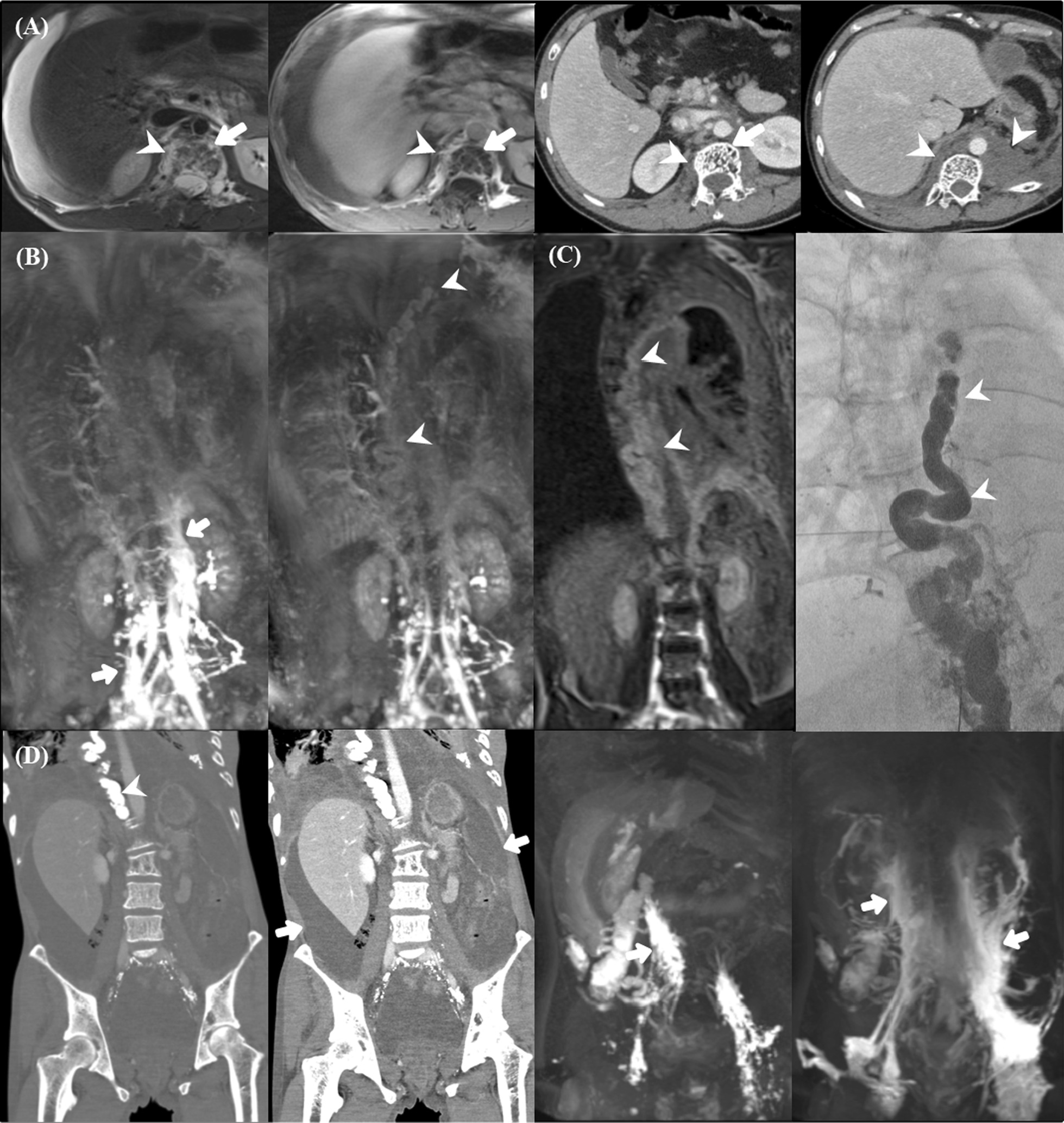
A 31-year-old male with Gorham–Stout disease. A 31-year-old man diagnosed with Gorham‒Stout disease presented with dyspnea related to massive chylous pleural effusion on the right side. Dynamic contrast-enhanced magnetic lymphangiography (DCMRL) consisted of axial T2-weighted image (T2WI), axial/coronal contrast-enhanced T1-weighted image (T1WI), and maximum intensity projection (MIP) reconstruction images. **A** T2WI (first column) and contrast-enhanced T1WI (second column) reveal multiple bundles of hypertrophied lymphatic vessels with a dark signal in the thickened right posterior parietal pleura and contrasting bright signals (arrowheads), and there is a contrast-enhancing vertebral lesion (arrows). Contrast CT (third and fourth columns) showed osteolytic changes (arrow) and retroperitoneal soft-tissue lesions (arrowheads). **B** MIP images (first and second columns) showing extensive collateral lymphatic vascular channels in the retroperitoneal space (arrows) and a faint giant thoracic duct (arrowheads). **C** A faintly filled, large abnormal thoracic duct is visible on the coronal T1WI before MIP reconstruction (first column, arrowheads). The giant thoracic duct is confirmed using digital subtraction angiography (second column, arrowheads). **D** After thoracic duct embolization (first column, arrowheads), the right pleural effusion was controlled, but uncontrolled ascites developed (second column, arrows), and the patient returned to the hospital one year later. When DCMRL was performed again, lymphatic flow (third and fourth columns, arrows) in the abdominal cavity was enriched, which was thought to have occurred because of the blocked outlet

Non-osseous involvements included lung parenchymal lesions in four patients and pleural involvement in four patients. Imaging findings of the lung parenchymal lesions included diffuse increased interstitial opacities, bronchovascular bundle thickening, and diffuse pleural thickening of the bilateral hemithorax (Fig. 1B). Splenic lesions were identified in four patients whose MRIs demonstrated multiple round cystic lesions with decreased signal intensity on T1-weighted images and increased signal intensity on T2-weighted images (Fig. 1C). 13 patients (86.7%) had infiltrative soft-tissue abnormalities adjacent to the osseous involvement. The soft-tissue lesions were seen as an ill-defined, low-attenuation stranding adjacent to the osseous changes on CT images (Figs. 2 and 3A).

**Fig. 4:**
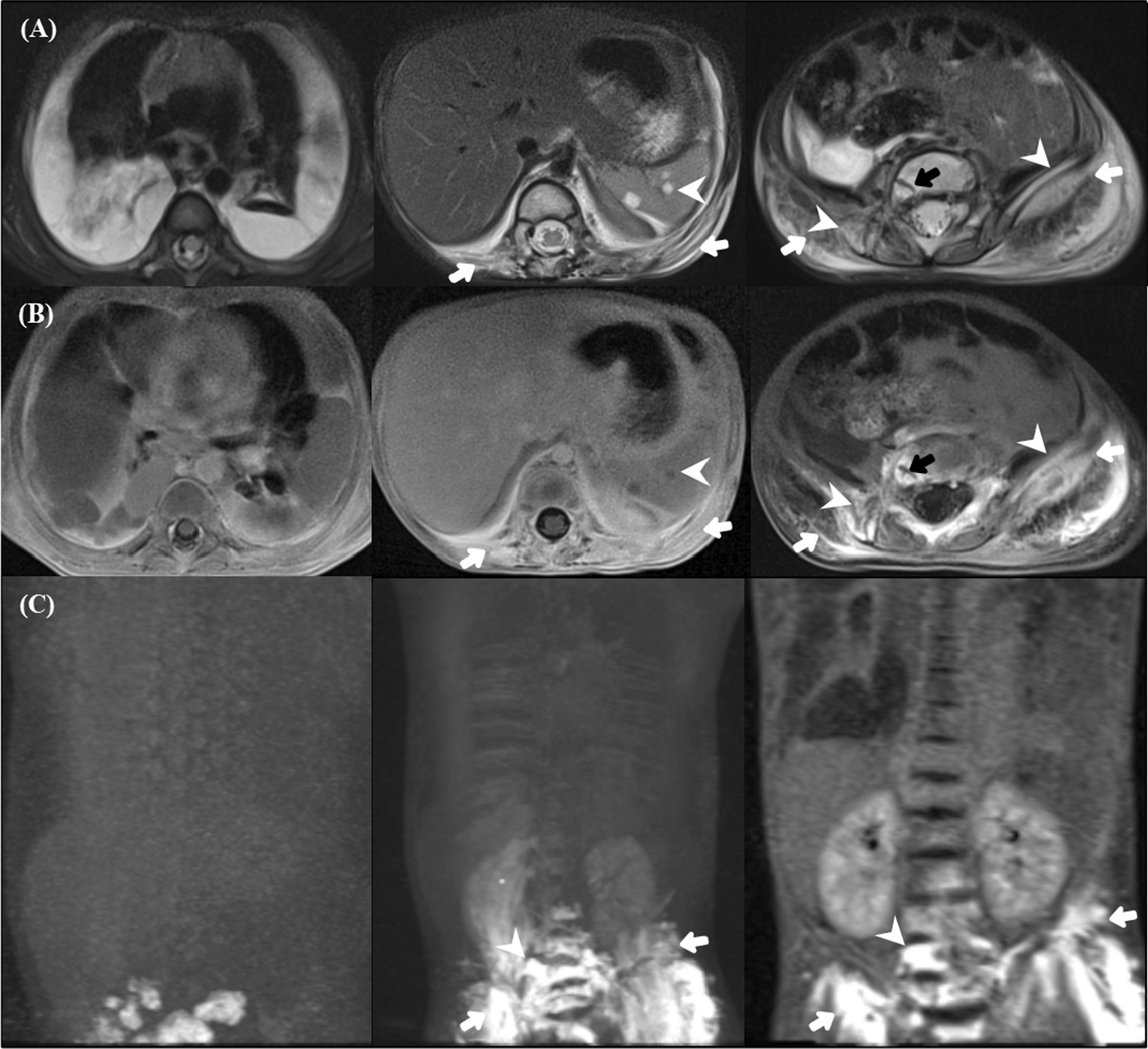
A 3-year-old female with Gorham–Stout disease. A 3-year-old female patient diagnosed with Gorham‒Stout disease presented with asymmetric buttock volume. Dynamic contrast-enhanced magnetic lymphangiography (DCMRL) consisted of axial T2-weighted image (T2WI), axial/coronal contrast-enhanced T1-weighted image (T1WI), and maximum intensity projection (MIP) reconstruction images. **A** There is a large volume of pleural effusion (first column), small volume of ascites, splenic cysts (second column, arrowhead), soft-tissue infiltration (second and third columns, white arrows), and bone lesions (third column, arrowheads) on T2WI. **B** There is bony enhancement in the ileum (third column, arrowheads) and vertebrae (black arrow), and soft-tissue thickening with enhancement (second and third columns, arrows). **C** MIP showing an abundantly enhanced retroperitoneal lymphatic system and soft tissue (arrows) and enhanced vertebrae (arrowheads), but the cisterna chyli and thoracic duct were not visible

Among 15 patients in our study, four patients underwent DCMRL examinations. T2-weighted images of all patients showed pleural effusion. DCMRL findings are presented in Additional file 1: Table S2. The peri- and intraosseous lesions were enhanced through DCMRL, which involves injecting a contrast agent through the inguinal lymph nodes (Figs. 2B, 3A, 4A). All patients showed abnormal collateral lymphatic proliferation in the peri-osseous soft tissues, chest/abdominal wall, or retroperitoneal space. Peri-osseous soft-tissue lesions were also visualized at the level of the right posterior pleura, which could be the leading cause of the chylous pleural effusion, and some of them involved the adjacent iliac bone (Figs. 2B, 3A, 4B). DCMRL revealed weak central conducting lymphatic flow with abundant collateral flow in all patients (Figs. 2C, 3B, 4C). The thoracic ducts were visualized in three patients, but two of them had abnormal dilatation of the giant tortuous thoracic duct (Figs. 2D and 3C). The thoracic duct embolization was performed successfully in three patients who showed direct contrast leakage or reflux into the thoracic cavity on DCMRL. In contrast, one patient underwent medical treatment without an interventional procedure because of the non-visualization of the direct cause of the symptom.

Among seven patients with chylous pleural effusions, one patient (patient no. 10) died, another (no. 7) was a follow-up loss, and five surviving patients received medical treatment with sirolimus, propranolol, and pamidronate after thoracic duct embolization. All patients achieved radiologically stable conditions concerning the chylothorax (mean ± standard deviation of follow-up duration: 3.6 ± 0.6 years) after treatment. Still, one patient underwent additional lymphovenous creation surgery due to severe leg edema.

## Discussion

GSD, also known as vanishing bone disease or massive osteolysis, is a rare lymphatic malformation of unknown etiology [13]. Progressive osteolysis can affect single or multiple bones by progressive destruction of the osseous matrix due to an imbalance of osteoclasts and osteoblasts, as well as proliferation of endothelial cells in lymphatic structures [14]. Therefore, when considering the pathogenesis of GSD, imaging of the lymphatic vessels is essential to accurately assess the diagnosis, disease extent, and treatment response. This study summarizes the clinical history, osseous involvement, and extraosseous manifestations of GSD. In particular, the recently introduced imaging modality, DCMRL, was used to assess the disease based on its mechanism, despite the small number of patients.

The clinical manifestations of GSD vary widely depending on the site and extent of involvement [15]. GSD results in pain, swelling, weakness, and pathological fractures of the affected bones or adjacent areas. Therefore, conventional plain radiography focused on assessing osseous involvement of the spine, pelvis, lower extremities, and ribs. Osteolytic lesions in GSD are associated with local lymphatic vessel proliferation. In this study cohort, the most affected sites were the spine (73.3%), pelvic bone (60.0%), lower extremities (40.0%), and ribs (40.0%), as seen on conventional plain radiography. Despite the variance of bone lesions depending on the stage, plain radiographs can provide the most significant clues for diagnosing GSD and can be helpful for accurately assessing the range of bone destruction.

However, lymphatic vessel proliferation can occur anywhere; thus, extraosseous involvement can lead to chylous effusion in the pleural or abdominal cavity, resulting in poor prognosis and life-threatening symptoms such as dyspnea and infection [16–18]. This study also revealed chylothorax in seven patients (46.7%) and chylous ascites in one patient. There was a relatively high incidence of edematous changes in the trunk and lower extremities (60.0%). These changes might be related to the high rate of sepsis history, such as pneumonia or cellulitis (80.0%). Conventional MRI can help assess the extent of non-osseous involvement, such as lung, pleura, spleen, and soft-tissue changes [19].

Considering the mechanisms that drive osteolysis and lymphangiogenesis in GSD, an accurate assessment of lymphatic vessels might be needed in patients with GSD. DCMRL is an emerging imaging tool for evaluating lymphatic anatomy and physiologically assessing the lymphatic flow. Compared to the previous lymphatic imaging modalities, such as lymphoscintigraphy or lipiodol lymphangiography, DCMRL has better spatial resolution and more physiologic lymphatic flow due to non-oily contrast agents. To our knowledge, there are few reports on DCMRL findings in patients with GSD. DCMRL, as a one-stop imaging modality, can be used to evaluate non-osseous involvement and lymphatic flow. DCMRL can reveal the phenomenon of abnormal propagation of contrast agents from the inguinal lymph nodes through the central conducting lymphatics and dermal backflow along the chest and abdominal walls. It can directly show the proliferation of lymphatic vessels within the bone in patients with GSD, which are useful imaging findings that cannot be seen on plain radiographs or other imaging modalities. All patients who underwent DCMRL in this study presented with altered anatomical lymphatics and functional flow with collateralization. In particular, the giant thoracic duct seen in two patients with GSD may be a tortuous dilated thoracic duct deformed due to weak central conducting lymph flow, and DCMRL data collection about the giant thoracic duct would be required to determine whether it is one of the characteristic findings in GSD patients. Furthermore, DCMRL could help determine further treatment so that interventional treatment such as embolization could proceed. Therefore, evaluation through DCMRL might be necessary and crucial to assess patients with GSD.

GSD may often be misdiagnosed as a neoplasm, tuberculosis, or chronic osteomyelitis owing to its rarity and unique clinical characteristics [20]. Therefore, a combination of clinical history, radiologic findings, and histopathological examination is the gold standard for the diagnosis of GSD. This study is useful in that it has shown in several cases that it is possible to directly view and evaluate lymphatic vessel proliferation using DCMRL in patients with GSD. However, because GSD is an extremely rare disease, this study has a limitation in that it investigated only a relatively small number of patients. Since all patients who underwent interventional embolization were also treated with sirolimus and pamidronate, it is difficult to know what the actual treatment effect was due to. There has yet to be a consensus on the most effective treatment for GSD. The treatment options for GSD patients consists of the mTOR inhibitor sirolimus and bisphosphonates such as pamidronate [21]. The effect of sirolimus blocks the expression of vascular endothelial growth factor as an mTOR inhibitor, which shows anti-angiogenic results in patients with GSD because of stopping the proliferation of lymphatic vessels [22]. Bisphosphonate agents are also reported as one of the medical treatment drugs because of their anti-osteoclastic and anti-angiogenic effects [21–23]. Based on the pathogenesis and previous case reports, our medical treatment protocol for patients with GSD adopted sirolimus and pamidronate for anti-osteoclastic and anti-angiogenic effects under off-label use. However, a randomized controlled trial is still needed to select the optimal medical treatment method.

## Conclusion

In conclusion, DCMRL imaging and plain radiography are very useful in determining the extent of GSD and are essential for its diagnosis. DCMRL is an essential imaging tool for the visualization of abnormal lymphatics in patients with GSD, which helps in further treatment. Therefore, in patients with GSD, it might be necessary to obtain not only plain radiographs but also MRI and DCMRL images, which might be an added valuable prognostic tool for patients with soft tissue extension beyond the typical osseous involvement.

## Supplementary Information

**Additional file 1.** **Supplementary Table S1.** Distribution of Gorham–Stout Disease Involvement. **Supplementary Table S2.** DCMRL characteristics in Gorham–Stout disease.

## Data Availability

Data sharing does not apply to this article as no datasets were generated or analyzed during the current study.

## Abbreviations

GSD: Gorham–Stout disease
DCMRL: Dynamic contrast-enhanced magnetic resonance lymphangiography
MRI: Magnetic resonance imaging
CT: Computed tomography
MIP: Maximum intensity projection
