# Supplementary material for: The spectrum of imaging manifestations of Gorham–Stout disease: a novel dynamic contrast-enhanced MR lymphangiography
Source: Orphanet J Rare Dis. 2023 Apr 26;18:96. doi: 10.1186/s13023-023-02704-7 (PMC10131433; doi:10.1186/s13023-023-02704-7)
Supplement: Supplementary file 1 — Additional file 1. Supplementary Table S1. Distribution of Gorham–Stout Disease Involvement. Supplementary Table S2. DCMRL characteristics in Gorham–Stout disease. [file 13023_2023_2704_MOESM1_ESM.docx]

**Supplementary Table S1.** Distribution of Gorham–Stout Disease Involvement

| **Involvement** | **Number (%)** |
| --- | --- |
| Osseous |  |
| Vertebra | 11 (73.3) |
| Cervical | 0 (0) |
| Thoracic | 7 (46.7) |
| Lumbar | 10 (66.7) |
| Sacral | 4 (26.7) |
| Above diaphragm | 8 (53.3) |
| Skull | 2 (13.3) |
| Rib | 6 (40.0) |
| Scapula | 1 (6.7) |
| Upper extremities | 3 (20.0) |
| Below diaphragm | 9 (60.0) |
| Iliac bone | 9 (60.0) |
| Lower extremities | 6 (40.0) |
| Non-osseous |  |
| Above diaphragm | 7 (46.7) |
| Lung Septal thickening | 4 (26.7) |
| Pleural thickening | 4 (26.7) |
| Mediastinal soft-tissue lesion | 2 (13.3) |
| Below diaphragm | 6 (40.0) |
| Splenic cysts | 4 (26.7) |
| Retroperitoneum | 2 (13.3) |
| Soft tissue | 13 (86.7) |
| Head and neck | 1 (6.7) |
| Chest wall | 6 (40.0) |
| Abdominal wall | 4 (26.7) |
| Buttock | 3 (20.0) |
| Scrotal swelling | 4 (26.7) |
| Extremities | 4 (26.7) |

**Supplementary Table S2.** DCMRL characteristics in Gorham–Stout disease

| **No.** | **Age (yr)** | **Central Conducting Lymphatics** | **Collateral Lymphatics** | **Flow into Osseous Structures** | |
| --- | --- | --- | --- | --- | --- |
| 1 | 3 | Not visible | Abdominal wall, Buttock | Yes (Vertebra, Ileum) |  |
| 2 | 14 | Giant thoracic duct with weak flow | Abdominal/Chest wall | Yes (Vertebra, Ileum) |  |
| 3 | 15 | Normal-looking thoracic duct | Thoracic duct branches, Retroperitoneum | Yes (Vertebra, Ileum) |  |
| 10 | 31 | Giant thoracic duct with weak flow | Retroperitoneum | Yes (Vertebra) |  |
